# The Use of Metabolomics to Elucidate Resistance Markers against Damson-Hop Aphid

**DOI:** 10.1007/s10886-018-0980-y

**Published:** 2018-07-06

**Authors:** Anna K. Undas, Florian Weihrauch, Anton Lutz, Rob van Tol, Thierry Delatte, Francel Verstappen, Harro Bouwmeester

**Affiliations:** 1 Laboratory of Plant Physiology, Wageningen University, Droevendaalsesteeg 1, 6708 PB Wageningen, The Netherlands; 2 Present Address: RIKILT, Wageningen University & Research, Akkermaalsboss 2, 6708 WB Wageningen, The Netherlands; 3 Bavarian State Research Center for Agriculture (LfL), Institute for Crop Science and Plant Breeding, Hop Research Center Huell, Wolnzach, Germany; 4 Wageningen Plant Research, Biointeractions and Plant Health, Droevendaalsesteeg 1, 6708 PB Wageningen, The Netherlands; 5 Present Address: Swammerdam Institute for Life Sciences, Plant Hormone Biology group, University of Amsterdam, Science Park 904, 1098 XH Amsterdam, The Netherlands

**Keywords:** Hop metabolites, Damson-hop aphid, Plant resistance, Plant defense, Untargeted metabolite profiling

## Abstract

*Phorodon humuli* (Damson-hop aphid) is one of the major pests of hops in the northern hemisphere. It causes significant yield losses and reduces hop quality and economic value. Damson-hop aphid is currently controlled with insecticides, but the number of approved pesticides is steadily decreasing. In addition, the use of insecticides almost inevitably results in the development of resistant aphid genotypes. An integrated approach to pest management in hop cultivation is therefore badly needed in order to break this cycle and to prevent the selection of strains resistant to the few remaining registered insecticides. The backbone of such an integrated strategy is the breeding of hop cultivars that are resistant to Damson-hop aphid. However, up to date mechanisms of hops resistance towards Damson-hop aphids have not yet been unraveled. In the experiments presented here, we used metabolite profiling followed by multivariate analysis and show that metabolites responsible for hop aroma and flavor (sesquiterpenes) in the cones can also be found in the leaves, long before the hop cones develop, and may play a role in resistance against aphids. In addition, aphid feeding induced a change in the metabolome of all hop genotypes particularly an increase in a number of oxidized compounds, which suggests this may be part of a resistance mechanism.

**Electronic supplementary material:**

The online version of this article (10.1007/s10886-018-0980-y) contains supplementary material, which is available to authorized users.

## Introduction

Hop is an indispensable raw material for brewing beer and is almost exclusively used by the brewing industry, in order to impart increased microbiological stability, to strengthen foam stabilization, and to improve taste and aroma of the product (Nance and Setzer 2011). The annual worldwide production of hop is approximately 100,000 metric tons on average (Barth-Haas Group 2016), which underlines the worldwide economic significance of this globally traded crop. Until recently, the general goal of hop breeding programs was to obtain cultivars high in yield and in α-acids content. Since the first decade of the twenty-first century, the interest of hop breeding programs has shifted to the development of new flavours, which are increasingly demanded by craft brewers. Surprisingly, the objective to develop new disease- and insect-resistant accessions, is currently of minor interest to hop breeders (Čerenak et al. 2009; Darby and Campbell 1996; Kralj et al. 1998; Seigner et al. 2005, 2009), notwithstanding hop is cultivated in monoculture, thus the risk of pest and disease outbreaks is increased. Several pathogens and pests are able to damage the hop plants significantly, thus causing serious yield losses, and sometimes the complete destruction of a crop (Mahaffee et al. 2009). In the northern hemisphere the Damson-hop aphid *Phorodon humuli* (Schrank) is a major pest of hop. It is an obligate holocyclic/heteroecious aphid with four species of *Prunus* − sloe, damson, plum, and cherry plum − serving as primary (winter) hosts and hop as its sole secondary (summer) host (Eppler 1986). It can also cause significant loss of yield, but even light infestations of the cones can already compromise their quality and reduce their economic value (Barber et al. 2003; Weihrauch et al. 2012).

Farmers control *P. humuli* with insecticides, although due to environmental concerns the number of registered active ingredients is steadily decreasing. The pesticide selection pressure often results in the development of resistant aphid genotypes (Hrdý et al. 1986). An integrated approach to pest management in hop is therefore badly needed in order to break this cycle and to prevent the selection of strains resistant to the few remaining registered insecticides. One cornerstone of such an integrated strategy consists in the breeding of hop cultivars that are, partially, resistant to *P. humuli*. Differences in varietal susceptibility to the hop aphid have been demonstrated previously (Campbell 1983; Darby and Campbell 1988; Dorschner and Baird 1988; Kralj et al. 1998; Weihrauch and Moreth 2005), but the mechanisms underlying these resistances are unknown. To cope with herbivorous insects, plants have evolved various and complex mechanisms of defence. In principle, two categories can be distinguished: constitutive and induced defence, which can be further sub-divided into direct and indirect defence. The first barrier is mechanical protection against insect attacks, such as the presence of hairs or a thick cuticle. However, the chemistry of plants plays a much more important role in defence/resistance mechanisms than these mechanical barriers (Smith and Boyko 2007; Züst and Agrawal 2016). Although extensively studied, the attractiveness, repellence and resistance mechanisms of plants against aphids are far from being fully unraveled (Dogimont et al. 2010; Miles 1999; Smith and Boyko 2007; Walling 2008; Züst and Agrawal 2016). In general aphids select their host plants based on plant colour and odor via complicated, multiple stage processes (Döring 2014; Mehrparvar et al. 2014; Pitino and Hogenhout 2012; Powell and Hardie 2001; Powell et al. 2006). Hop is very rich in secondary metabolites such as bitter acids (α-acids), prenylflavonoids and terpenoids. These families of molecules may play a role in host selection by aphids (Kryvynets et al. 2008). Farag et al. (2012a, b) using LC-MS and NMR techniques, managed to identify and quantify 46 hop metabolites, including 18 bitter acids, 12 flavonoids, three terpenes, three fatty acids and two sugars. Nance and Setzer (2011) and Yan et al. (2017) analysed the volatile components extracted from hop cones. Some of these compounds like β-myrcene, germacrene D and α-selinene were demonstrated to be not only important for hop aroma but also to play a role in resistance or susceptibility to hop powdery mildew (Čerenak et al. 2009). The bracts of the cones contain most of the polyphenols, while the terpenoids and bitter compounds are contained in the lupulin, a yellow sticky resin secreted by glands in the cones (Biendl et al. 2014). However, as the winged *P. humuli* morphs migrate to hop as their summer host long before the cones have been formed, the leaf metabolites are more likely explaining host selection behavior during the aphid’s spring migration. Plant metabolomics, a powerful tool, first described in 2000 (Fiehn et al. 2000) was developed to identify biomarkers responsible for metabolic characteristics and revealing metabolic mechanisms. In literature we can find that the tool was successfully used for example to enhance understanding of the mechanisms of induced defense response in rice plant against *C. suppressalis* (Liu et al. 2016) or to identify secondary plant compounds involved in hot plant resistance (Leiss et al. 2010). Additionally, metabolomics or metabolite profiling was used to study foxglove aphids (*Aulacorthum solani* Kaltenbach) on leaves of soybean (Sato et al. 2013), or to explain variation in acceptance or discrimination between plant species of pea aphid (Hopkins et al. 2017)

The present study objective was to identify key features of the leaf metabolome that correlated with *P. humuli* resistance (or susceptibility) in a broad panel of hop genotypes. For this purpose we used GC-MS metabolic profiling of apolar secondary metabolites in hop leaves before and after *P. humuli* infestation. The hop genotypes selected for this study were verities already used commercially or important parental lines with a full spectrum of resistance. Firstly, the resistance level of 20 field-grown genotypes was compared with the metabolic profile of leaves collected in early summer, when plants had not yet been colonized by aphids, therefore representing the possible constitutive defence compounds. Secondly, we assessed the metabolites induced by aphid feeding on six genotypes covering the full range of resistance. This second experiment was performed at two different time points in a controlled environment. Taken together, our results show that sesquiterpenes and lupulon contribute to the metabolic differentiation of hop genotypes and show a good correlation with resistance or sensitivity to aphids.

## Methods and Materials

### Selection of Plant Material and Growing Conditions

Plant material, used for metabolite profiling experiments, was grown in 2012 (experimental hop field) and in 2011 (greenhouse) in the facilities of the Hop Research Center Hüll, the Bavarian State Research Center for Agriculture, in Wolnzach, Bavaria, Germany. In both cases, field and JOCE-D-18-00011R greenhouse, the growing conditions were not controlled. Table 1 presents the list of 21 hop genotypes that were used, with additional information on their resistance level, as explained below. Twenty of these genotypes can be classified as European hop, while 2006/268/001 (vS1) is taxonomically recognized as pertaining to the *H. lupulus* var. *pubescens*, native to the mid-west of North America.

**Table 1:** List of hop cultivars and breeding lines that were used in this study

No	Code	Hop genotypes	Experiment	Infestation level in 2011^*a*^	Resistance ^*b*^
1	vS1	2006/268/001	field	8	very susceptible
2	vS2	93/010/036	field	7	very susceptible
3	PA	cv. Polaris	field	8	very susceptible
4	vS3	2002/186/740	field	9	very susceptible
5	HM	cv. Hallertauer Magnum	field, greenhouse	8	very susceptible
6	TU	cv. Hallertauer Taurus	field	8	very susceptible
7	HS	cv. Herkules	field, greenhouse	8	very susceptible
8	PE	cv. Perle	field	7	susceptible
9	OL	cv. Opal	field	8	susceptible
10	SR	cv. Saphir	field	7	susceptible
11	SD	cv. Smaragd	field	8	susceptible
12	iR1	2005/034/022	greenhouse	5	intermediate resistant
13	iR2	2004/011/028	field	5	intermediate resistant
14	iR3	2002/186/038	field	4	intermediate resistant
15	WH49	WH 49	field	4	intermediate resistant
16	HT	cv. Hallertauer Tradition	field	5	intermediate resistant
17	SE	cv. Spalter Select	field, greenhouse	3	intermediate resistant
18	R1	2002/185/004	field	3	resistant
19	R2	2002/186/735	field	3	resistant
20	R3	3 W 42–30-38	field, greenhouse	0	resistant
21	BO	cv. Boadicea	field, greenhouse	0	resistant

^a^0 = no aphid infestation, 1 = very little infestation: 1–4 aphids/leaf, no cone infestation, 3 = marginal infestation: 5–20 aphids/leaf, very little cone infestation, 5 = middle infestation: 20–100 aphids/leaf, visible cone infestation, 7 = heavy infestation: 100–1000 aphids/leaf, impeded cone formation, 9 = extreme infestation: > 1000 aphids/leaf, no cone formation

^b^Resistance categorization according to multiple-year observations of aphid infestation levels on the respective genotypes by hop breeder Anton Lutz

The hop genotypes belong to different Damson-hop aphid resistance groups as indicated in column “Resistance”. The column “Experiment” shows if the hop genotype was used in the greenhouse or the field experiment

The following genotypes were used:

- very susceptible (**vS**) breeding lines (2006/268/001 [vS1], 93/010/036 [vS2], 2002/186/740 [vS3]) and cultivars (Polaris [PA], Hallertauer Magnum [HM], Hallertauer Taurus [TU], Herkules [HS]),
- susceptible (**S**) cultivars (Perle [PE], Opal [OL], Saphir [SR], Smaragd [SD]),
- intermediate resistant (**iR**) breeding lines (2005/034/022 [iR1], 2004/011/028 [iR2], 2002/186/038 [iR3]) and cultivars (WH49 [WH49], Hallertauer Tradition [HT], Spalter Select [SE]),
- aphid-resistant (**R**) breeding lines (2002/185/004 [R1], 2002/186/735 [R2], 3 W 42–30-38 [R3]), and cultivar Boadicea [BO].

### Hop Genotype Classification into Resistance Categories

In 2011, so before the metabolite profiling experiments, the aphid infestation on the hop genotypes used in this study was monitored and quantified in an experimental hop field and used to categorise the hop genotypes into resistance classes, as follows (also see Table 1).

- 0 = no aphid infestation
- 1 = very little infestation: 1–4 aphids/leaf, no cone infestation
- 3 = marginal infestation: 5–20 aphids/leaf, very little cone infestation
- 5 = middle infestation: 20–100 aphids/leaf, visible cone infestation
- 7 = heavy infestation: 100–1000 aphids/leaf, impeded cone formation
- 9 = extreme infestation: > 1000 aphids/leaf, no cone formation

For the present study, these six resistance levels were reduced to four groups (very susceptible, susceptible, intermediate resistant and aphid-resistant), as shown in Table 1.

### Experimental Set-up

A schematic overview of the experimental setup is shown in the Supplementary Materials, Fig. S1. The plants analyzed in this study were grown under two different conditions. Firstly, 20 hop genotypes were grown in an experimental hop field (Wolnzach, Bavaria, Germany). These plants were free of aphids and were used to analyse the constitutive leaf metabolome, without aphid infestation. Secondly, to study the effect of aphids on the leaf metabolome, six genotypes were grown under greenhouse conditions and infested with *P. humuli* at two relevant time points for the plant-insect interaction.

For the former experiment, the plant material consisted of two (vS1, vS3, iR3, R1, R2), six (vS2, PA, TU, PE, OL, SR, SD, HT) and 12 (HM, HS, iR2, WH49, SE, R3, BO) root cuttings per hop genotype. Plants (leaves) were harvested 36 days after planting. Each genotype was analysed as four (vS1, vS3, iR3, R1, R2) or six (remaining genotypes) replicates, where 10–13 leaves per replicate were harvested, immediately frozen in liquid nitrogen, ground to a fine powder with a mortar and pestle, and stored at −80 °C until analysis. Prior to harvesting, all genotypes were carefully investigated for the presence of aphids on leaves and aphid infestation could be excluded in all cases.

For the aphid infestation experiment, six representative hop genotypes were chosen that significantly vary in their resistance against aphids (HM, HS, iR1, SE, R3, BO). At the beginning of the experiments (early and later summer), plants (60 pots per treatment) were divided into two groups, one marked as control (^c^) and second as treatment (^t^) and kept apart to avoid cross-contamination. In early summer, plants were grown for 47 days and leaves of plants marked as treatment were exposed to aphid infestation (*n* = 5 per leaf) 13 days prior to harvest. In late summer, plants were grown for 45 days and leaves of plants marked as treatment were exposed to aphid infestation (*n* = 20 per leaf) 10 days prior to harvest. From each plant four comparable leaves without (control) or with aphids (treatment group) were collected. Prior to harvest all leaves of the treatment group were cleaned from aphids with a paintbrush. Leaves from five plants were combined to one replicate, immediately frozen in liquid nitrogen, ground to a fine powder with a mortar and pestle, and stored at −80 °C until analysis (five replicates per treatment were collected).

### Chemical Analysis

For GC-MS analysis, 500 mg (FW) of the frozen ground leaf material was extracted twice with 1 mL of dichloromethane (Sigma-Aldrich, The Netherlands) containing 4.92 mM (*Z*)-nerolidol (Sigma-Aldrich, The Netherlands) as internal standard. The material was then sonicated for 15 min in an ultrasonic water bath (3510 Branson, USA) and centrifuged for 10 min at 1609 ×g (Harrier 15/80, MSE, United Kingdom). The dichloromethane extracts were dried through anhydrous sodium sulfate (Sigma-Aldrich, The Netherlands).

Samples were analysed on an Agilent 7890A gas chromatograph connected to the 5795C mass selective Triple-Axis Detector (Agilent Technologies, United States). For that purpose, 1 μl of extract was injected at 250 °C in splitless mode on a ZB-5MS column (Phenomenex, 30 m × 0.25 mm; ID 0.25 μm) with 5 m guard column with a constant flow of helium at 1 mL/min. The oven was programed for 1 min at 45 °C and then subsequently ramped at 10 °C/min to 300 °C, at which it was kept for 7 min. The ionization potential was set at 70 eV, and scanning was performed from 45 to 450 amu, with a scanning speed of 3.99 scans/s.

### Data Processing and Statistical Analysis

The GC-MS data from all experiments were baseline corrected and aligned using Metalign (Lommen 2012) followed by mass/peak filtering using METOT (https://wiki.nbic.nl/index.php/METOT), an in-house developed web-based tool. Filtering was based on the observation of a mass/peak in at least three samples (minimal number of replicates) and a peak height above a certain threshold value considered being the noise level (selected empirically).

The obtained filtered data were clustered using MSClust (Tikunov et al. 2012), yielding a matrix with sample names in columns and putative metabolites (named centrotypes) in rows. The matrix consisting of the corresponding peak heights was then normalized to 1 mg FW leaf material and to the peak height of the IS (relative abundances). The MS spectra of the centrotypes that were reconstructed by MSClust were compared with the NIST14 spectral library and in house MS spectra/retention index libraries.

The data (relative abundances of centrotypes) from all chemical analyses were first analysed with *1-way ANOVA* with *Bonferroni* correction (*P* < 0.05) to select the metabolites that show significant differences between treatments. Next, exploratory multivariate analyses (principal component analysis, *PCA* and hierarchical cluster analysis, *HCA*) were performed first with GeneMaths XT (ver. 2.12, Applied Maths NV, Belgium), followed by Multibase: Excel Add-ins for PCA and PLS (ver. 2015, Numerical Dynamics) and MetaboAnalyst 3.0 (Xia et al. 2015). Canonical redundancy analysis (*RDA*) was performed with Canoco for windows 4.5 and/or Canoco5 software (http://www.canoco5.com). Prior to *PCA*, *HCA* and *RDA*, data were normalized by median value, log2 transformed and auto scaled. *HCA* analysis was performed using Pearson clustering and the complete aggregation procedure. The *RDA* analysis was performed using four resistance groups as environmental factors. For the analysis of aphid infested data, a multivariate classification method, partial least squares - discriminant analysis (*PLS-DA*) on two groups (control and treatment) was performed with MetaboAnalyst 4.0. Data were pre-processed as for the exploratory multivariate analyses.

## Results

### The GC-MS Metabolome of Hop Leaves

**Fig. 1:**
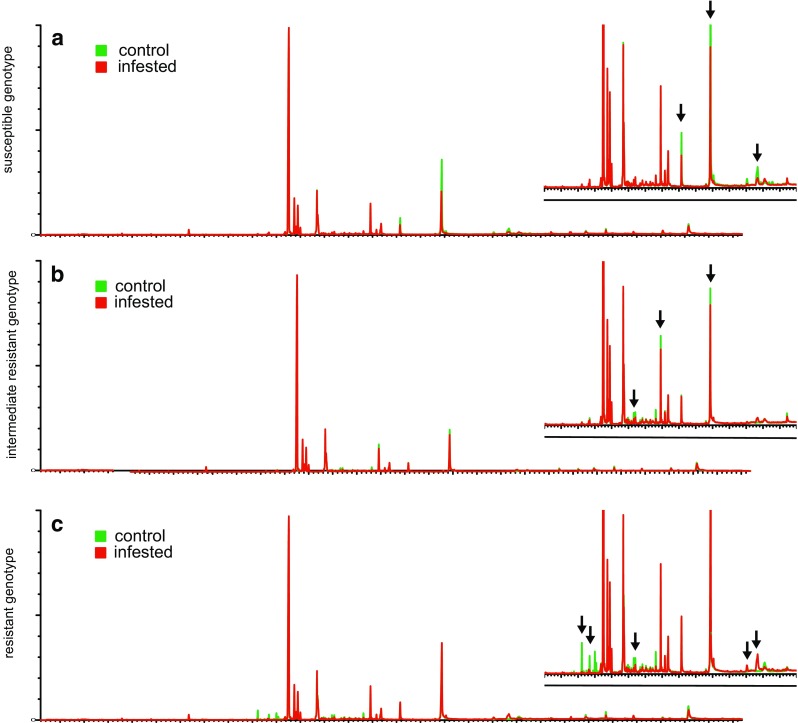
GC-MS chromatograms (time vs. relative abundance) of representative hop genotypes (a,b,c, susceptible, intermediate resistant and resistant genotypes respectively). Chromatograms of control (green) and Damson-hop aphid infested (red) genotypes are shown. Arrows indicate a number of the compounds that changed upon aphid infestation

Based on the aphid counting in the field, as summarized in Table 1, the hop genotypes used in the present study were divided into three arbitrary categories: aphid susceptible or very susceptible (S, vS), intermediate resistant (iR) and aphid resistant (R) genotypes. We investigated the compounds responsible for the different level of resistance in the leaves by analyzing the dichloromethane extracts with GC-MS. Figure 1 shows the metabolite profiles (chromatograms) of hop plants belonging to the three mentioned resistance categories (a-c respectively), with (red lines, “infested”) or without (green lines, “control”) aphid feeding. Aphid feeding resulted in the increase or suppression of one or a number of metabolites in the attacked plants. Qualitative assessment of the data showed that the profile of R genotypes changed more than that of iR or S plants when they were exposed to aphid feeding, and that those changes are associated with more than one metabolite. Therefore, taking into account the complexity of the data set, an untargeted metabolomics approach was further used to identify metabolites related with aphid-resistance/susceptibility.

### Metabolic Profile of 20 Field-Grown Genotypes

During the field experiment leaf samples for 20 genotypes were analysed using untargeted metabolomics. This untargeted approach allowed for the detection of 213 putative metabolites. The *1-way ANOVA* with *Bonferroni* correction showed that there was a significant difference (*P* < 0.05) between the analysed hop samples in 176 mass-signals. After filtering out the noise, 97 mass-signals (metabolites) remained, of which many could be putatively annotated (Table S1). These were used in the subsequent multivariate analyses to identify the relevant differences between the samples.

**Fig. 2:**
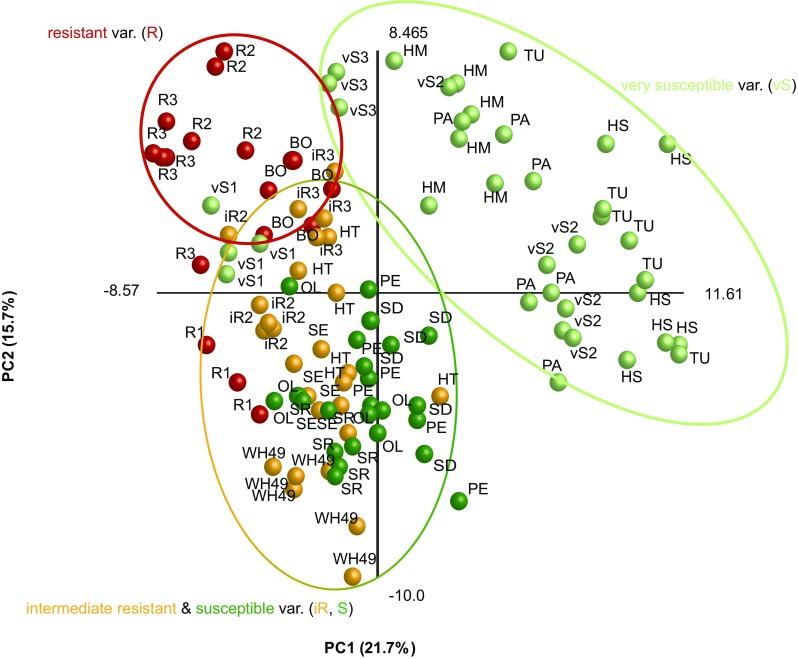
*PCA* scores plot representing the metabolic profiles of 20 hop genotypes obtained through GC-MS analysis. Each of hop genotype is represented by 6 biological replicates with exception of genotypes vS1, vS3, iR3, R1 and R2 that are represented by 4 biological replicates. Very susceptible, susceptible, intermediate resistant and aphid-resistant genotypes are represented by light green (vS: vS1 – vS3, PA, HM, TU, HS), green (S: PE, OL, SR, SD), orange (iR: iR1 – iR3, WH49, HT, SE), and red (R: R1 – R3, BO) spheres respectively. A detailed description of the hop genotypes is provided in Table 1. The corresponding *PCA* loadings plot can be found in Supplementary materials Fig. S2

**Fig. 3:**
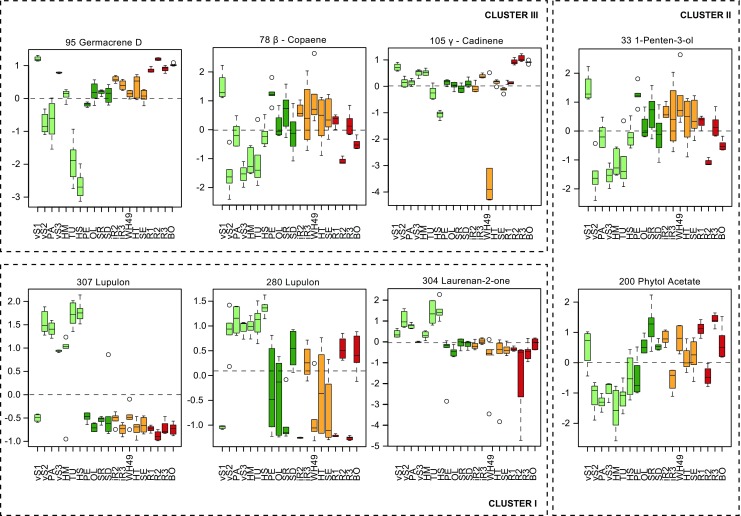
Relative abundances of a number of hop metabolites (n = 6, with exception of hop genotypes vS1, vS3, iR3, R1 and R3 where *n* = 4). The metabolite intensities were determined during the analysis of 20 hop genotypes by GC-MS. The metabolites were chosen based on their significance in the *PCA* analysis

From the principle component analysis (*PCA*) (Fig. 2) the vS plants were clearly separated as a distinct group (vS2, PA, vS3, HM, TU, and HS) with exception of the genotypes 2006/268/001 (vS1) and, to a lower extent, 2002/186/740 (vS3). The separation between the vS and other groups explained 21.7% (PC1) of the variation. At the same time PC2 (15.7%) differentiated further within S, iR and R genotypes. The variation between the biological replicates was larger for the vS (e.g. PA, HS) than for the S, iR and R genotypes (e.g. iR2, SE, R3, BO). This grouping was explained (the loadings plot, Fig. S2) by two main clouds of metabolites that cause the separation along PC1, and three weakly separated groups that cause separation along PC2 (Clusters I-III, Table 2). Along PC1, the largest group consisted of the sesquiterpenes (Cluster III), and compounds annotated as higher alkanes and phytol-like compounds (Cluster II), which are separated from sesquiterpenes along PC2. The remaining group (Cluster I) comprised lupulon-like compounds, sterols and their possible precursors. Sesquiterpenes (Cluster III) generally had higher amplitudes in the R (e.g. 95 germacrene D), and iR genotypes, with the exception of WH49 (e.g. 105 γ-cadinene), or were equally distributed among the analysed hop genotypes (e.g. 78 β-copaene) (Fig. 3). The lupulon-like compounds and sterols (Cluster I) were more defined and usually the vS genotypes had clearly higher levels of these compounds than the other ones (e.g. 307 lupulon, 280 lupulon, 304 laurenan-2-one) (Fig. 3). Alcohols, sterols and their precursors from Cluster II (e.g. 33 1-penten-3-ol, 200 phytol acetate) had the lowest concentration in vS and the highest in R genotypes.

**Table 2:** Composition of clusters that are responsible for trends that were found by *pca* based on 20 analysed hop genotypes

CLUSTER III				CLUSTER II				CLUSTER I			
	Annotation	MF	PC1		Annotation	MF	PC1		Annotation	MF	PC1
95	Germacrene D	949	−0.1804	201	11,12,13-Trinor-5-eudesmene	508	−0.19524	307	Lupulon	711	0.174065
80	Isogermacrene D	810	−0.1467	35	Malonic acid, methyl-, dimethyl ester	510	−0.19395	297	Cholest-4-en-3-one, 4-methyl-	585	0.171443
83	Valerena-4,7(11)-diene	754	−0.1411	33	1-Penten-3-ol	709	−0.19112	1	3-Methylbutanoic acid (Isovaleric acid)	900	0.167924
81	Valerena-4,7(11)-diene	900	−0.1382	23	Propanal	545	−0.18668	308	Stigmasterol	692	0.167913
76	β-Caryophyllene	974	−0.1375	393	γ-Sitosterol	941	−0.18108	287	Lupulon	567	0.167464
82	ε-Muurolene	855	−0.1354	4	3-Hexen-1-ol, (Z)-	953	−0.17804	274	Nabilone	610	0.166265
129	Germacrene D-4-ol	860	−0.1306	310	1-Docosene	885	−0.17131	281	unknown	<500	0.164262
69	β-Elemene	862	−0.1134	200	Phytol acetate	870	−0.16991	302	Stimast-5-en-3-ol, (3beta)- “beta-Sitosterol”	533	0.161709
103	Germacrene D	892	−0.1121	203	Neophytadiene	905	−0.16976	9	1-Pentanol, 4-methyl-	766	0.160088
90	4(15),5-Muuroladiene, cis- or Epi-bicyclosesquiphellandrene	808	−0.1038	197	Phytol acetate	909	−0.16487	282	Lupulon	632	0.159505
78	β-Copaene	925	−0.0878	252	γ-Decalactone, γ-methyl-	717	−0.16266	18	3-Penten-2-one	700	0.152649
159	ent-Germacra-4(15),5,10(14)-trien-1β-ol	927	−0.0877	368	1-Docosanol, acetate	913	−0.15414	303	Ergost-25-ene-3,5,6,12-tetrol, (3β,5α,6β,12β)-	614	0.151474
56	Bicycloelemene	899	−0.0871	232	Phytol	807	−0.15334	304	Laurenan-2-one	437	0.147864
70	Germacrene A/ß-Elemene	920	−0.0853	19	Octen-3-ol (1-)	836	−0.15066	272	unknown/Lupulon	674	0.147822
104	Germacrene D	690	−0.0783	371	Octadecane	831	−0.14775	3	5-Hexen-3-ol	827	0.14664
372	α-Tocopherol ‘Vitamin E’	874	−0.0771	363	Nonadecane	882	−0.14591	280	Lupulon	869	0.144679
8	3-Methyl-3-butenenitrile ‘Methallyl cyanide’	703	−0.0694	191	2-Dodecenal, (E)-	692	−0.14507	296	Lupulon	661	0.140196
97	Bicyclogermacrene	927	−0.0679	163	Farnesol, (E,E)-	631	−0.14506	273	unknown	<500	0.134837
160	Δ-Cadinene	549	−0.0657	108	Shyobunol	691	−0.1421	301	Lupulon	690	0.132991
113	β-Bazzanene	610	−0.0625	166	Furan, 2-(12-tridecynyl)- “Avocadynofuran”	498	−0.13731	298	a-Homocholest-4a-en-3-one	554	0.132744
77	γ-Elemene	920	−0.0576	162	Cyclohexanol, 3-ethenyl-3-methyl-2-(1-methylethenyl)-6-(1-methylethyl)-, [1R-(1α,2α,3β,6α)]-	958	−0.13279	293	unknown	<500	0.129641
117	Germacrene B	861	−0.0573	246	Hexadecane	869	−0.12718	133	Humulene epoxide II	898	0.124545
106	elema-1,3-dien-6α-ol	705	−0.0522	375	Benzoic acid, isobutyl ester	728	−0.12058	279	Lupulon	786	0.10964
154	α-Cadinol	631	−0.0519	374	unknown	<500	−0.1196				
151	α-Muurolol ‘Torreyol’	809	−0.0515	397	β-Vetivone	539	−0.1192				
353	Octadecane	751	−0.0471	355	1-Docosanol, acetate	921	−0.11374				
114	Elemol	852	−0.0443	285	Docosane	824	−0.10444				
131	p-Menth-1(7)-en-9-ol	626	−0.0442	369	Isovaleric acid, tetracosyl ester	810	−0.10111				
115	Elemol	845	−0.044	364	Butyl 2-methylbutanoate	519	−0.08535				
93	ar-Curcumene	857	−0.0278	359	Isovaleric acid, tetracosyl ester	782	−0.08507				
89	alpha-Humulene	953	−0.0171	231	unknown	529	−0.07987				
105	γ-Cadinene	909	−0.0149	357	Stigmasterol	579	−0.07027				
				283	1-Pentadecanol	532	−0.06569				
				13	2(5H)-Furanone, 5,5-dimethyl-	886	−0.05972				
				358	unknown	<500	−0.05818				
				395	Carissone	515	−0.05441				
				153	Isogeranial	678	−0.05198

**Fig. 4:**
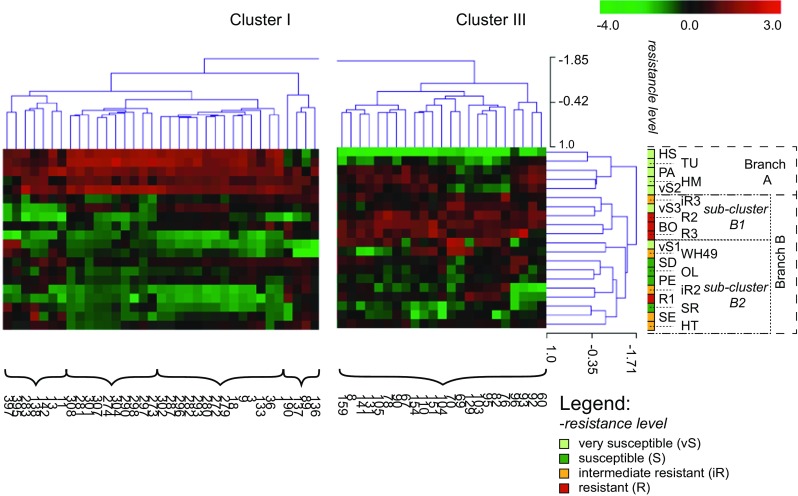
Simplified outcome (dendrograms) of the *HCA* representing the metabolites detected thought GC-MS analysis of 20 hop genotypes (n = 6, with exception of hop genotypes vS1, vS3, iR3, R1 and R3). The horizontal axis of the dendrograms represents the distance or dissimilarity between clusters of the metabolites (Cluster I and Cluster III, Cluster II not shown), while the vertical axis represents the clusters of genotypes (Branch A and Branch B consists of sub-clusters B1 and B2). The detected metabolites are represented by numbers. Detailed information about the metabolites is provided in Supplementary materials Table S1

The link between above mentioned metabolites and aphid resistance was further analysed using hierarchical cluster analysis (*HCA*), which highlights the similarity between different hop samples based on the metabolites they contain. *HCA* analysis clustered samples into two main groups, coined Branch A and Branch B, with two sub-clusters B1 and B2 (Fig. 4). Branch A contained vS hop genotypes, with exception of vS1 and vS3. Branch B consisted of three out of four R hop genotypes, vS3, iR3 (sub-cluster B1), and vS1, S, remaining iR and R1 hop genotypes (sub-cluster B2).

This clustering was caused by three groups of metabolites (Cluster I and III Fig. 4; Cluster II shown in supplementary materials Fig. S3). The most R genotypes (R2, R3, BO), but also vS (vS2, PA, HM, TU) were characterized by higher levels of metabolites (Cluster III), which were annotated as sesquiterpenes (C15H24) or sesquiterpene alcohols (C15H26O or C15H24O). The vS genotypes (vS2, PA, HM, TU, HS) had high levels of compounds clustered into Cluster I. Metabolites of this cluster were in many cases annotated as humulene, humulene oxides, and plant sterols. Cluster II (Fig. S3) represented metabolites, which were relatively abundant in the S and iR genotypes, and were belonging to the longer chain hydrocarbons. In summary, *PCA* and *HCA* analyses allowed to distinguish between hop genotypes based on their leaf metabolome, and to identify that the sesquiterpenes were present in higher levels in the R genotypes.

**Fig. 5:**
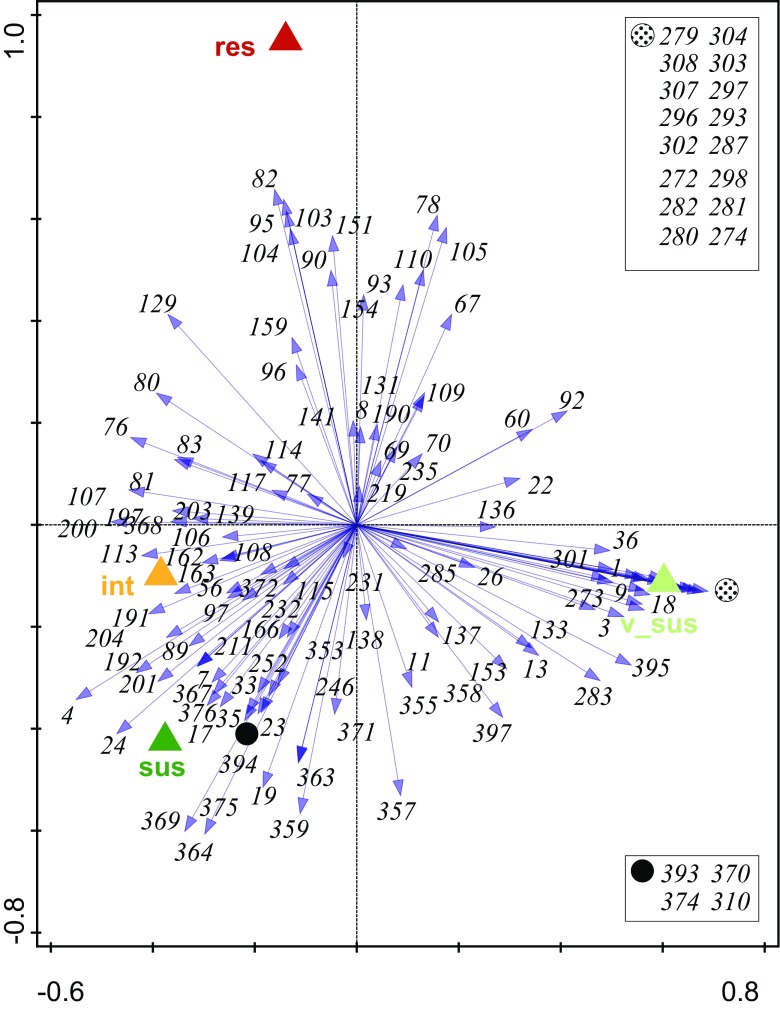
Schematic representation of the *RDA* diagram that is based on 20 analysed hop genotypes and aphid resistance level (*n* = 6, with exception of hop genotypes vS1, vS3, iR3, R1 and R3). Arrows represent metabolites that are responsible for the observed differences between resistant, tolerant and (very) susceptible hop genotypes (encoded as res, tol, v_sus and sus, respectively). Metabolites that overlap are indicated by the solid black () and dotted () symbols and are indicated in the Figure. The detected metabolites are represented by a number; detailed information about the metabolites is provided in Supplementary materials Table S1

To narrow down individual metabolites that correlate with the resistance, redundancy analysis (*RDA*) was performed. *RDA* was carried out using resistance level as an environmental variable (explanatory variable). The first dimension (AX1) separated the vS hop genotypes from the rest (Fig. 5). The second dimension (AX2) separated the genotypes into two groups: iR, (v)S, and R genotypes. Closer analysis of responsible metabolites that were ranked in order of theirs positions on the ordination axes showed that the sesquiterpenes ε-muurolene and germacrene D (82, 95/103 respectively) drive the separation of the R genotypes. On the other side of the plot, compounds annotated as lupulon or lupulon-like compounds (e.g. 280, 282, 272, 287) seemed to drive the separation of the vS genotypes. The separate clustering of the iR and S genotypes was driven by higher levels of hydrocarbons and hydrocarbon alcohols or esters such as green leaf volatiles, e.g. isovaleric acid tetracosyl ester, butyl 2-methylbutanoate, (*Z*)-3-hexen-1-ol and (*Z*)-3-hexen-1-ol acetate (369, 364, 4, 24). In summary, we identified metabolic features showing good correlation with resistance in a field experiment performed with 20 diverse hop genotypes. However, this experiment explained only the constitutive defences observed in the plants, in the next step we have investigated the molecules that upon induction would correlate with hop resistance to aphids.

### Aphid Induced Metabolite Changes

Based on the results obtained from the field study, a sub-selection of hop genotypes was further investigated in the greenhouse setting. The aim of this experiment was to detect metabolite changes that occur after aphid infestation, and to identify metabolites that may be involved in induced defence mechanisms. Hereto, the metabolite profile of hop leaves of six varieties; HM, HS, iR1, SE, R3 and BO was investigated for aphid infection, 4 weeks after planting (Fig. S1). When the infestation experiment was repeated in late summer (Fig. S1 and Fig. S4), no clear metabolite changes occurred upon aphid infestation (see below). As the objective of this study was to determine metabolite changes as a response to aphid spring migration we focus on the spring experiment.

Untargeted GC-MS analysis allowed for the detection of 215 putative metabolites, of which 109 displayed a significant difference between the varieties (*P* < 0.05, *1-way ANOVA* with *Bonferroni* correction). The subset of significantly different metabolites was used as an input for untargeted statistical analysis.

**Fig. 6:**
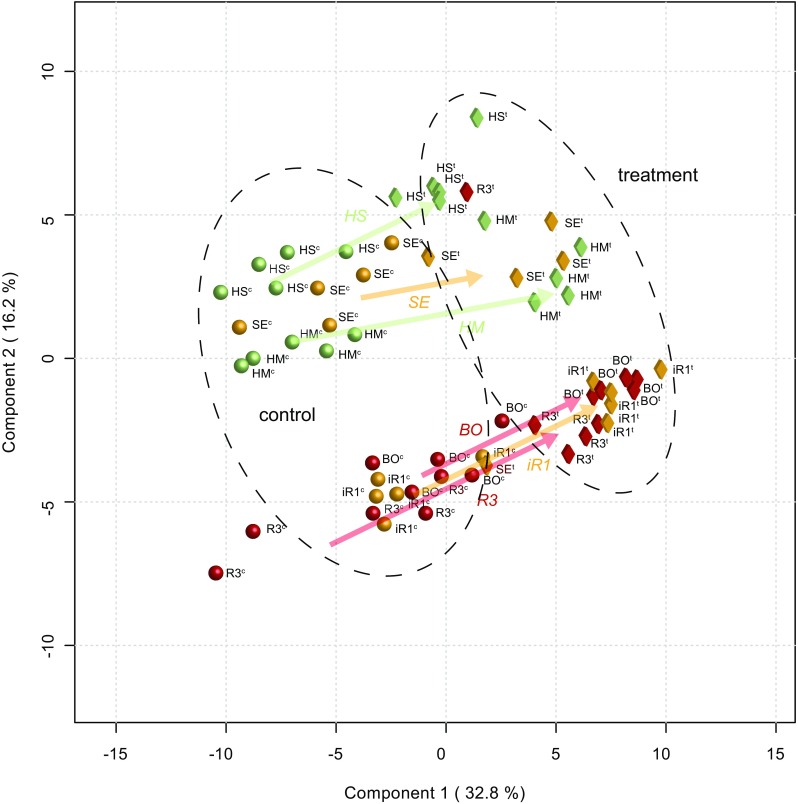
*PLS-DA* scores plot representing the metabolic profiles, obtained through GC-MS analysis, of 6 hop genotypes induced by aphid infestation (number of biological replicates n = 5). The control hop genotypes are represented by spheres (HM^c^, HS^c^, iR1^c^, SE^c^, R3^c^, BO^c^), while infested by aphid genotypes are represented by diamonds (HM^t^, HS^t^, iR1^t^, SE^t^, R3^t^, BO^t^). Shifts represented by arrows illustrate the changes in the metabolite composition between the control and infested (treated) state. The hop genotypes are also coded according to their resistance level: green, yellow and red reflect S, iR and R, respectively. A detailed description of the hop genotypes is provided in Table 1. The corresponding *PLS-DA* loadings plot can be found in Supplementary materials Fig. S4

**Fig. 7:**
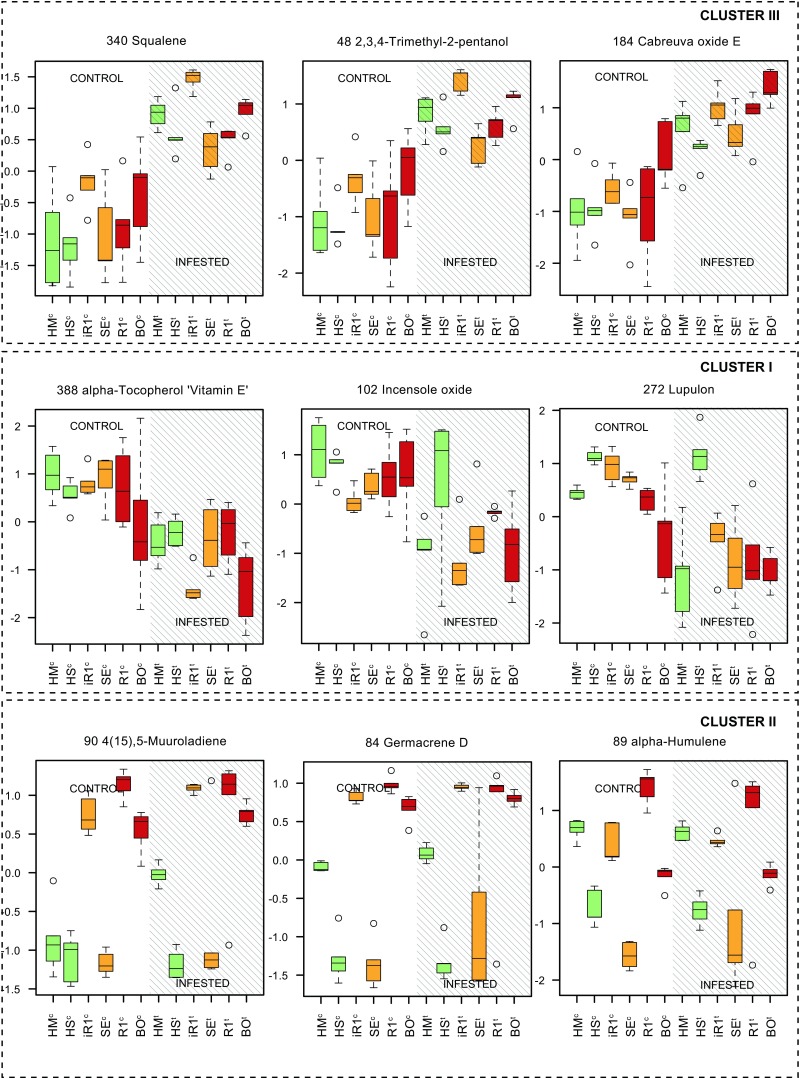
Relative abundances of selected hop metabolites (*n* = 5). The metabolite intensities were determined during the GC-MS analysis of hop genotypes infested by aphids. The metabolites were chosen based on their importance in *PLS-DA*

The results of the supervised *PLS-DA* analysis between two (control and treatment) groups are shown in Fig. 6 (Fig. 6 shows scores plot, Fig. S5 shows loadings plot). The *PLS-DA* scores plot (component 1 explains 32.8%, component 2 explains 16.2% of total variation) shows that the first component clearly separates the control plants from the treated (infested) hop genotypes (Fig. 6). The component 2 divides the hop genotypes into two well distinguished groups, containing S (HS, HM) and R (BO, R3), while iR genotypes are divided between these two groups. The corresponding loadings plot displayed three dense clouds of metabolites (Fig. S5); clusters I, II and III along loadings 1, and clusters II and I/III along loadings 2. Clusters I and III contain compounds with high VIP score values (a measure of a variable’s importance in the PLS-DA model) that influence the separation into control and infested genotypes. Cluster II contains compounds annotated as sesquiterpenes, with very low VIP score values, indicating that these compounds do not play a role in the induced resistance mechanisms. Analysis of the first 30 metabolites with the highest VIP score values showed that 22 of these were higher in infested genotypes (Cluster III) than in the respective controls (Cluster I) (Table 3). Figure 7 shows the data for a number of these compounds, where e.g. abundances of squalene and cabreuva oxide E (340 and 184, respectively) were higher, while abundances of α-tocopherol and lupulon (388 and 272, respectively) were lower in infested genotypes. A second *PLS-DA* of control and aphid infested plants, which was performed on data collected in late summer (Fig. S4) did not show strong shifts between control and aphid-induced plants. In general control genotypes were grouped together with treated ones, with the exception of the S genotypes. Also in this experiment, however, the main resistance groups separated well. Moreover, the separation between genotypes iR1, R3, BO on the one hand and HS, HM, SE on the other was stronger than in early spring.

**Table 3:** Composition of clusters that are responsible for trends found in *pls-da* based on control and infested (treatment) hop genotypes (vip scores)

CLUSTER III				CLUSTER I				CLUSTER II			
	Annotation	MF	VIP score		Annotation	MF	VIP score		Annotation	MF	VIP score
340	Squalene	895	1.83	388	α - Tocopherol	893	1.50	90	4(15),5-Muuroladiene, cis-	785	0.28
333	unk	<500	1.82	44	Artemisyl acetate	708	1.44	70	Germacrene A	834	0.22
48	Acetamide	656	1.82	329	unk	<500	1.35	151	α-Muurolol	809	0.16
337	unk	<500	1.81	102	Incensole oxide	645	1.32	93	ar-Curcumene	857	0.15
335	unk	<500	1.79	11	3-Heptanol, 4-methyl-	572	1.29	120	α-Cubebene	753	0.12
328	unk	<500	1.77	20	6-Methyl-5-hepten-2-one	745	1.27	67	α-Copaene	860	0.12
184	Cabreuva oxide E	684	1.73	272	Lupulon	674	1.22	84	Germacrene D	828	0.09
175	Cedroxyde	792	1.68	318	Lupulon	807	1.22	96	α-Zingiberene	891	0.08
152	Nerolidol, (Z)-	743	1.66					105	γ-Cadinene	909	0.06
172	Sesquicineol-2-one	649	1.64					401	Silphiperfol-6-ene	551	0.06
179	α-Bisabololoxide C	663	1.63					92	γ-Muurolene	844	0.04
173	Nerolidol, (Z)-	742	1.61					95	Germacrene D	949	0.02
3	5-Hexen-3-ol	773	1.61					89	α-Humulene	952	0.01
177	Lilac alcohol	621	1.56					76	β-Caryophyllene	954	0.01
169	β-Bazzanene	615	1.55					99	α-Muurolene	778	0.01
180	Incensole oxide	638	1.53								
170	Farnesol, (2E,6E)-	682	1.50								
45	p-Menth-3-ene	719	1.48								
168	Sesquicineol-2-one	636	1.41								
182	3-Nonen-2-one	507	1.36								
195	unk	<500	1.29								
13	2(5H)-Furanone, 5,5-dimethyl-	886	1.28

## Discussion

Damson-hop aphid is one of the major pests of hop in the northern hemisphere. Even light infestations of the harvested cones can already damage their quality and reduce their economic value. An integrated approach to pest management focusing on the breeding of hop cultivars at least partially resistant to *P. humuli*, as well as methods to screen for such resistance, would be very desirable. The use of untargeted metabolomics has contributed to the elucidation of resistance mechanisms in two *Senecio* species and tomato to Western flower thrips (Leiss et al. 2009; Mirnezhad et al. 2010), and in soybean to foxglove aphid (Sato et al. 2013). However, to our knowledge this approach has not yet been used to study resistance in hops. In this study we have used metabolomics to pinpoint leaf metabolites that correlate with, and are possibly responsible for, hop resistance against *P. humuli*. Because aphids colonise their host long before the cones have been formed (Born 1968), we decided to focus on the metabolite composition of leaves, as it would add important knowledge to the existing data on cone metabolites. By using multivariate analysis in two independent experiments, we show that aphid-resistant genotypes have higher levels of a number of sesquiterpenes.

The results of the untargeted *PCA* of 20 hop genotypes showed that based on metabolite composition hop genotypes cluster in groups with different resistance levels (Fig. 2). Only the breeding line vS1 did not follow this pattern. However, in contrast to the other genotypes we investigated, the breeding line vS1 has the North American *H. lupulus* var. *pubescens* in its pedigree, contrary to all other tested genotypes that were bred from European or East Asian genotypes. Even if pubescence has been associated with insect resistance this was not the case for vS1 (Hanley et al. 2007). Weaker differences between S and iR hop genotypes are supported by the work of Weihrauch et al. (2013), in which the iR cultivar SE grouped either with S or iR genotypes, depending on the season when the assessment was performed. The closer analysis of responsible metabolites by *RDA* showed that the sesquiterpenes, ε-muurolene and germacrene D (82 and 95/103, respectively), drive the separate clustering of the R genotypes. Both compounds have been shown to play a role in plant-aphid interaction (Bruce et al. 2005; Saad et al. 2015). Our results are supported by Čerenak et al. (2009), who showed a link between essential oil compounds of hop cultivars and their resistance/susceptibility to Powdery mildew. Among the markers that they reported, were santalene, germacrene D, α-selinene, and caryophyllene epoxide. In our study, germacrene D was strongly correlated with aphid resistance. Additionally, Kralj et al. (1998) recognized three markers correlating with hop resistance to aphids, where two compounds were positively annotated as α- and β-pinene. In our study these metabolites were not found, which can be explained by the fact that Kralj et al. (1998) studied the plant headspace, while we analysed hop leaf extracts. Next, we have identified compounds annotated as lupulon or lupulon-like compounds that seem to drive the separation of the vS genotypes. A high beta acid content of hop leaves has been reported to be a good indicator for aphid susceptibility (Kammhuber 1997). In contrast, these components of the beta acid family of leaf-specific bitter acids have repellent and oviposition-deterring effects on two-spotted spider mites *Tetranychus urticae*, another well-known pest of hop (Jones et al. 1996, 2010).

Plant can activate specific defences to deal with pests, the so called induced defences. The current study also examined the influence of aphid feeding on the hop leaf metabolome, to pinpoint the induced metabolites upon aphid infestation. Aphid feeding induced leaf metabolome changes in all hop genotypes. Various classes of molecules were up or down regulated (Table 3). However, this effect was only observed in spring and not in late summer, when the experiment was repeated. These findings are consistent with those of Weihrauch et al. (2013) and suggest that late stage resistance might be driven by another resistance mechanism. The elevated levels of oxidized compounds such as cabreuva oxide E, cedroxyde or sesquicineol-2-one upon aphid infestation would suggest the influence of those metabolites in the spring resistance mechanism, while lupulon-like and α-tocopherol-like compounds were reduced upon infestation. Lupulon has been shown to influence the behavior of aphids on hop and to change between early and late summer (Kryvynets et al. 2009).

In summary, in this study two different experiments were performed. One concentrated on constitutive resistance mechanisms and showed that resistant genotypes contain higher amounts of sesquiterpenes; this class of metabolites could be used as a constitutive resistance marker. The second experiment investigated the induced resistance mechanisms and indicated that oxidized compounds such as e.g. sesquicineol-2-one are more important than sesquiterpenes.

With this study we have narrowed down the group of metabolites that are of importance in hop resistance mechanisms towards Damson-hop aphid. Further confirmation with Damson-hop aphid feeding experiments will be needed. However, in conjunction with the hop genome and the understanding of the biosynthesis of sesquiterpenes and bitter acids (Cattoor et al. 2013; Clark et al. 2013; Hill et al. 2017), it should be possible to design and test specific molecular markers related to the molecules we identified here.

## Electronic supplementary material

ESM 1 (XLSX 41.2 kb)

ESM 2 (PDF 154 kb)

ESM 3 (PDF 173 kb)

ESM 4 (PDF 1.04 mb)

ESM 5 (PDF 10.6 mb)

ESM 6 (PDF 989 kb)

## References

[CR1] Barber A, Campbell CAM, Crane H, Darby P, Lilley R (2003). Cost-benefits of reduced aphicide usage on dwarf hops susceptible and partially resistant to damson-hop aphid. Ann Appl Biol.

[CR2] Barth-Haas Group (2016). Der Barth-Bericht Hopfen 2015/2016.

[CR3] Biendl M, Engelhard B, Forster A, Gahr A, Lutz A, Mitter W, Schmidt R, Schönberger C (2014). Hops: their cultivation, composition and usage.

[CR4] Born M (1968). Beitrage zur Bionomie von *Phorodon humuli* (Schrank, 1801). Archiv für Pflanzenschutz.

[CR5] Bruce TJ, Birkett MA, Blande J, Hooper AM, Martin JL, Khambay B, Prosser I, Smart LE, Wadhams LJ (2005). Response of economically important aphids to components of *Hemizygia petiolata* essential oil. Pest Manag Sci.

[CR6] Campbell CAM (1983). Antibiosis in hop (*Humulus lupulus*) to the damson-hop aphid, *Phorodon humuli*. Ent Exp & Appl.

[CR7] Cattoor K, Dresel M, De Bock L, Boussery K, Van Bocxlaer J, Remon J-P, De Keukeleire D, Deforce D, Th H, Heyerick A (2013). Metabolism of hop-derived bitter acids. J Agric Food Chem.

[CR8] Čerenak A, Kralj D, Javornik B (2009). Compounds of essential oils as markers of hop resistance (*Humulus lupulus)* to powdery mildew (*Podosphaera macularis*). Acta agriculturae Slovenica.

[CR9] Clark SM, Vaitheeswaran V, Ambrose SJ, Purves RW, Page JE (2013). Transcriptome analysis of bitter acid biosynthesis and precursor pathways in hop (*Humulus lupulus*). BMC Plant Biol.

[CR10] Darby P, Campbell CAM (1988). Resistance to *Phorodon humuli* in hops. IOBC/WPRS Bulletin.

[CR11] Darby P, Campbell CAM (1996) Aphid-resistant hops - the key to integrated pest management in hops. Brighton Crop Protection Conference on Pests and Diseases 1996:893–898

[CR12] Dogimont C, Bendahmane A, Chovelon V, Boissot N (2010). Host plant resistance to aphids in cultivated crops: genetic and molecular bases, and interactions with aphid populations. C R Biol.

[CR13] Döring TF (2014). How aphids find their host plants, and how they don't. Ann Appl Biol.

[CR14] Dorschner KW, Baird CR (1988). Susceptibility of hop to *Phorodon humuli*. Entomol Exp Appl.

[CR15] Eppler A (1986). Untersuchungen zur Wirtswahl von *Phorodon humuli* Schrk. I. Besiedelte Pflanzenarten. Anzeiger für Schädlingskunde, Pflanzenschutz, Umweltschutz.

[CR16] Farag MA, Porzel A, Schmidt J, Wessjohann LA (2012). Metabolite profiling and fingerprinting of commercial cultivars of *Humulus lupulus* L. (hop): a comparison of MS and NMR methods in metabolomics. Metabolomics.

[CR17] Farag MA, Porzel A, Wessjohann LA (2012). Comparative metabolite profiling and fingerprinting of medicinal licorice roots using a multiplex approach of GC–MS, LC–MS and 1D NMR techniques. Phytochemistry.

[CR18] Fiehn O, Kopka J, Dörmann P, Altmann T, Trethewey RN, Willmitzer L (2000). Metabolite profiling for funcational genomics. Nat Biotechnol.

[CR19] Hanley ME, Lamont BB, Fairbanks MM, Rafferty CM (2007). Plant structural traits and their role in anti-herbivore defence. Perspect Plant Ecol Evol Syst.

[CR20] Hill ST, Sudarsanam R, Henning J, Hendrix D (2017) HopBase: a unified resource for *Humulus* genomics. Database, Volume 2017, bax009. https://doi-org.ezproxy.library.wur.nl/10.1093/database/bax009, 2017 10.1093/database/bax009 PMC5467566 28415075

[CR21] Hopkins DP, Cameron DD, Butlin RK (2017). The chemical signatures underlying host plant discrimination by aphids. Sci Rep.

[CR22] Hrdý I, Kremheller HT, Kuldová J, Lüders W, Ula J (1986). Insektizidresistenz der Hopfenblattlaus, *Phorodon humuli*, in böhmischen, bayerischen und baden-württembergischen Hopfenanbaugebieten. Acta Entomol Bohemoslov.

[CR23] Jones G, Campbell CAM, Pye BJ, Maniar SP, Mudd A (1996). Repellent and oviposition deterring effects of hop beta acids on the two-spotted spider mite *Tetranychus urticae*. Pest Manag Sci.

[CR24] Jones G, Campbell CAM, Hardie J, Pickett JA, Pye BJ, Wadhams LJ (2010). Integrated management of two-spotted spider mite *Tetranychus urticae* on hops using hop ß-acids as an antifeedant together with the predatory mite *Phytoseiulus persimilis*. Biocontrol Sci Tech.

[CR25] Kammhuber K (1997). Investigations about the contents of the lupulin glands of hop leaves and their importance for hop breeding. Monatsschrift für Brauwissenschaft.

[CR26] Kralj D, Kač M, Dolinar M, Žolnir M, Kralj S (1998) Marker-assisted hop (*Humulus lupulus* L.) breeding. Monatsschrift für Brauwissenschaft 1998:111–119

[CR27] Kryvynets O, Walker F, Zebitz CPW (2008). Einfluss der Bitterstoffe des Hopfens auf das Wirtswahlverhalten von *Phorodon humuli* (Schrank), Homoptera, Aphididae. Mitteilungen der Deutschen Gesellschaft für allgemeine und angewandte Entomologie.

[CR28] Kryvynets O, Walker F, Zebitz CPW (2009). Bitterness of hops during the growing season. Mitteilungen der Deutschen Gesellschaft für allgemeine und angewandte Entomologie.

[CR29] Leiss KA, Choi YH, Abdel-Farid IB, Verpoorte R, Klinkhamer PGL (2009). NMR metabolomics of Thrips (*Frankliniella occidentalis*) resistance in *Senecio* hybrids. J Chem Ecol.

[CR30] Leiss KA, Choi YH, Verpoorte R, Klinkhamer PGL (2010). An overview of NMR-based metabolomics to identify secondary plant compounds involved in host plant resistance. Phytochem Rev.

[CR31] Liu Q, Wang X, Tzin V, Romeis J, Peng Y, Li Y (2016). Combined transcriptome and metabolome analyses to understand the dynamic responses of rice plants to attack by the rice stem borer *Chilo suppressalis* (Lepidoptera: Crambidae). BMC Plant Biol.

[CR32] Lommen A, Hardy NW, Hall RD (2012). Data (pre-)processing of nominal and accurate mass LC-MS or GC-MS data using MetAlign. Plant metabolomics. Methods in molecular biology (methods and protocols).

[CR33] Mahaffee WF, Pethybridge SJ, Gent DH (2009) Compendium of hop diseases and pests. St. Paul: American Phytopathological Society, APS Press

[CR34] Mehrparvar M, Mansouri SM, Weisser WW (2014). Mechanisms of species-sorting: effect of habitat occupancy on aphids' host plant selection. Ecological Entomology.

[CR35] Miles PW (1999). Aphid saliva. Biol Rev.

[CR36] Mirnezhad M, Romero-González RR, Leiss KA, Choi YH, Verpoorte R, Klinkhamer PGL (2010). Metabolomic analysis of host plant resistance to thrips in wild and cultivated tomatoes. Phytochem Anal.

[CR37] Nance MR, Setzer WN (2011). Volatile components of aroma hops (*Humulus lupulus* L.) commonly used in beer brewing. Journal of Brewing and Distilling.

[CR38] Pitino M, Hogenhout SA (2012). Aphid protein effectors promote aphid colonization in a plant species-specific manner. MPMI.

[CR39] Powell G, Hardie J (2001). The chemical ecology of aphid host alternation: how do return migrants find the primary host plant?. Appl Entomol Zool.

[CR40] Powell G, Tosh CR, Hardie J (2006). Host plant selection by aphids: behavioral, evolutionary, and applied perspectives. Annu Rev Entomol.

[CR41] Saad KA, Roff MNM, Hallett RH, Idris AB (2015). Aphid-induced Defences in Chilli affect preferences of the whitefly, *Bemisia tabaci* (Hemiptera: Aleyrodidae). Sci Rep.

[CR42] Sato D, Akashi H, Sugimoto M, Tomita M, Soga T (2013). Metabolomic profiling of the response of susceptible and resistant soybean strains to foxglove aphid, *Aulacorthum solani* Kaltenbach. J Chromatogr B.

[CR43] Seigner E, Lutz A, Radic-Miehle H, Seefelder S, Felsenstein FG (2005) Breeding for powdery mildew resistance in hop (*Humulus* L.): strategies at the hop research center, Huell, Germany. Acta Hortic (668):19–29. 10.17660/ActaHortic.2005.668.1

[CR44] Seigner E, Lutz A, Oberhollenzer K, Seidenberger R, Seefelder S, Felsenstein F (2009) Breeding of hop varieties for the future. Acta Hortic (848):49–57. 10.17660/ActaHortic.2009.848.4

[CR45] Smith CM, Boyko EV (2007). The molecular bases of plant resistance and defense responses to aphid feeding: current status. Ent Exp & Appl.

[CR46] Tikunov Y, Laptenok S, Hall R, Bovy A, de Vos R (2012). MSClust: a tool for unsupervised mass spectra extraction of chromatography-mass spectrometry ion-wise aligned data. Metabolomics.

[CR47] Walling LL (2008). Avoiding effective defenses: strategies employed by phloem-feeding insects. Plant Physiol.

[CR48] Weihrauch F, Baumgartner A, Felsl M, Kammhuber K, Lutz A (2012). The influence of aphid infestation during the hop growing season on the quality of harvested cones. Brewing Science.

[CR49] Weihrauch F, Baumgartner A, Felsl M, Kneidl J, Lutz A (2013) Simple is beautiful: a new biotest for the aphid tolerance assessment of different hop genotypes. Acta Hortic (1010):97–102. 10.17660/ActaHortic.2013.1010.10

[CR50] Weihrauch F, Moreth L (2005). Behavior and population development of *Phorodon humuli* (Schrank) (Homoptera: Aphididae) on two hop cultivars of different susceptibility. J Insect Behav.

[CR51] Xia JG, Sinelnikov IV, Han B, Wishart DS (2015). MetaboAnalyst 3.0-making metabolomics more meaningful. Nucleic Acids Res.

[CR52] Yan D, Wong Y, Tedone L, Shellie R, Marriott P, Whittock S, Koutoulis A (2017). Chemotyping of new hop (*Humulus lupulus* L.) genotypes using comprehensive two-dimensional gas chromatography with quadrupole accurate mass time-of-flight mass spectrometry. J Chromatogr A.

[CR53] Züst T, Agrawal AA (2016). Mechanisms and evolution of plant resistance to aphids. Nature Plants.

