# Supplementary material for: The Use of Metabolomics to Elucidate Resistance Markers against Damson-Hop Aphid
Source: J Chem Ecol. 2018 Jul 6;44(7):711–26. doi: 10.1007/s10886-018-0980-y (PMC6096525; doi:10.1007/s10886-018-0980-y)
Supplement: Supplementary file 2 — (PDF 154 kb) [file 10886_2018_980_MOESM2_ESM.pdf]

	The set-up of experiments				
Location:	Greenhouse facilities		Greenhouse facilities		Field experiment
	CONTROL (^{c})	TREATMENT (^{t})	CONTROL (^{c})	TREATMENT (^{t})	
Period:<br>(1) start of exp.<br>(2) aphid infestation<br>(3) harvest	(1) 20th April 2011<br><br>(3) 4/5th June 2011	(1) 20th April 2011<br>(2) 24th May 2011<br>(3) 4/5th June 2011	(1) 27th June 2011<br><br>(3) 10th August 2011	(1) 27th June 2011<br>(2) 1st August 2011<br>(3) 10th August 2011	(1) 9th May 2012<br><br>(3) 13th June 2012
Replication:	4 leaves each plant<br>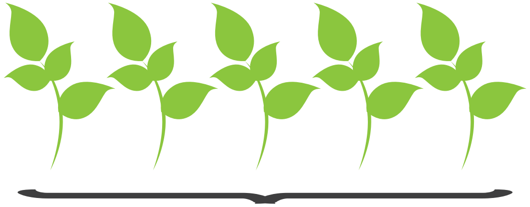<br>5 plants = 1 replicate		4 leaves each plant<br>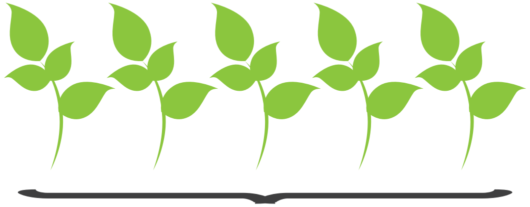<br>5 plants = 1 replicate		collected 10-13 leaves from 1 root stock:<br>(12 root stocks): HM, HS, T2, WH49, SE, R3, BO<br>(6 root stocks): vS2, PA, TU, PE, OL, SR, SD, HT<br>(2 root stocks): vS1, vS3, T3 R1, R2<br><br>1 root stock = 1 replicate
Nos of biological replicates:	5 replicates		5 replicates		4 replicates (vS1, vS3, T3, R1, R2)<br>6 replicates (other genotypes)
Used hop genotypes :	HM, HS, T1, SE, R3, BO		HM, HS, T1, SE, R3, BO		as mentioned above

Fig. S1 The set-up of the experiments performed in this study. The abbreviations for the hop genotypes are provided in Table 1.
