# Supplementary material for: The Use of Metabolomics to Elucidate Resistance Markers against Damson-Hop Aphid
Source: J Chem Ecol. 2018 Jul 6;44(7):711–26. doi: 10.1007/s10886-018-0980-y (PMC6096525; doi:10.1007/s10886-018-0980-y)
Supplement: Supplementary file 3 — (PDF 173 kb) [file 10886_2018_980_MOESM3_ESM.pdf]

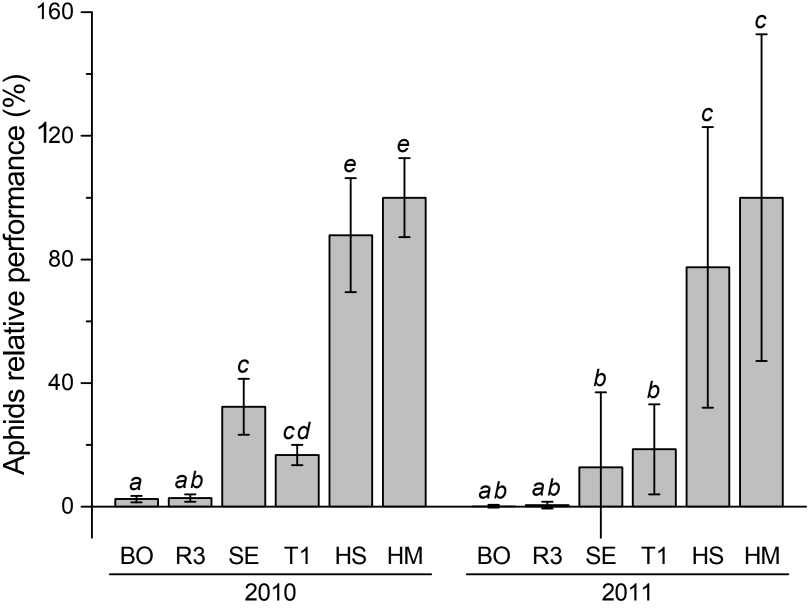

Fig. S2 An overview of Damson-hop aphid performance tests on different hop genotypes as performed by Weihrauch et al. (2013). The performance experiments were conducted in 2010 and 2011. Bars with different letters differ significantly according to post-hoc ANOVA ($P<0.05$). A detailed description of the hop genotypes is provided in Table 1.
