# Supplementary material for: The Use of Metabolomics to Elucidate Resistance Markers against Damson-Hop Aphid
Source: J Chem Ecol. 2018 Jul 6;44(7):711–26. doi: 10.1007/s10886-018-0980-y (PMC6096525; doi:10.1007/s10886-018-0980-y)
Supplement: Supplementary file 4 — (PDF 1.04 mb) [file 10886_2018_980_MOESM4_ESM.pdf]

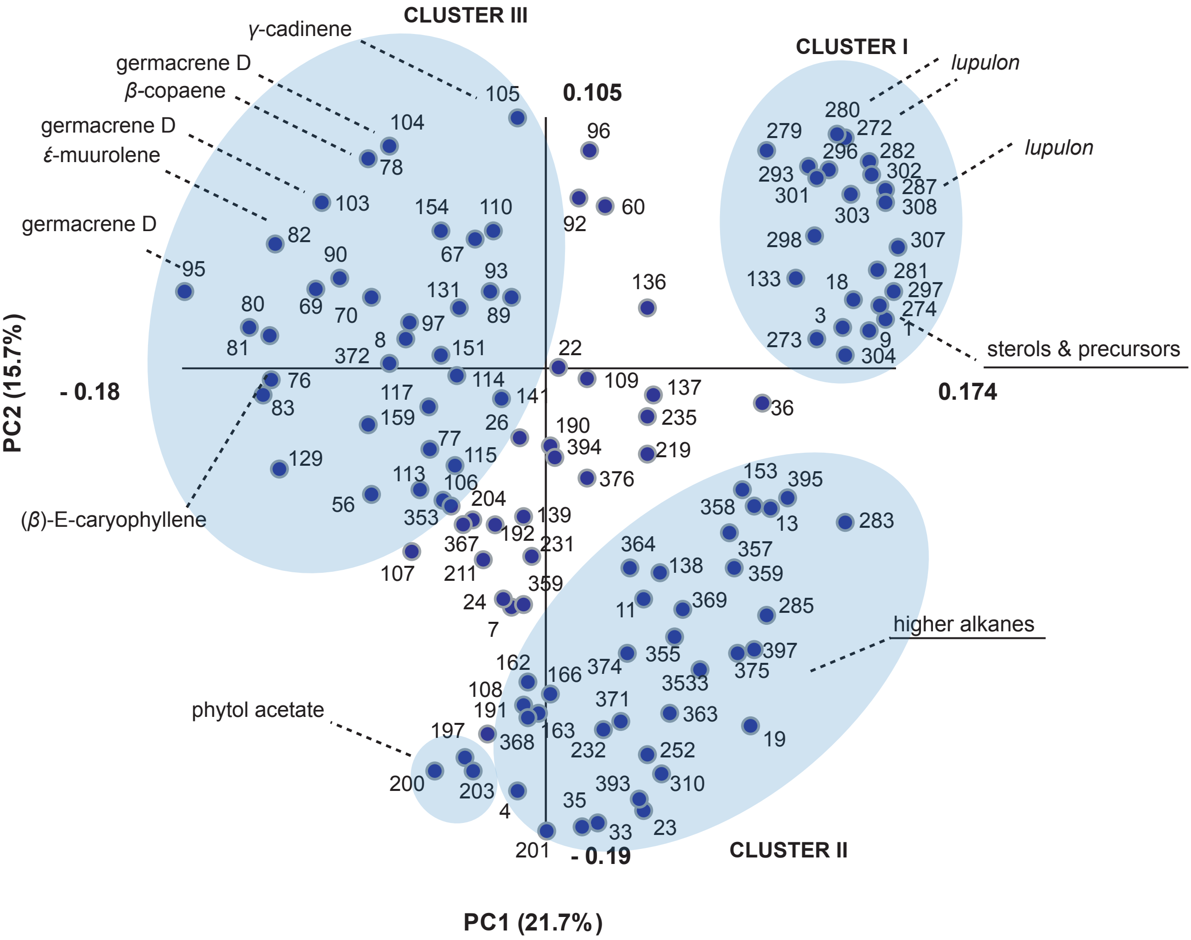

Fig. S3 PCA loadings plot representing the metabolic profiles of 20 hop genotypes obtained through GC-MS analysis. Metabolites that drive the separation of hop genotypes into different resistance groups are highlighted in blue. The corresponding PCA scores plot is shown in Fig. 2.
