# Supplementary material for: The Use of Metabolomics to Elucidate Resistance Markers against Damson-Hop Aphid
Source: J Chem Ecol. 2018 Jul 6;44(7):711–26. doi: 10.1007/s10886-018-0980-y (PMC6096525; doi:10.1007/s10886-018-0980-y)
Supplement: Supplementary file 5 — (PDF 10.6 mb) [file 10886_2018_980_MOESM5_ESM.pdf]

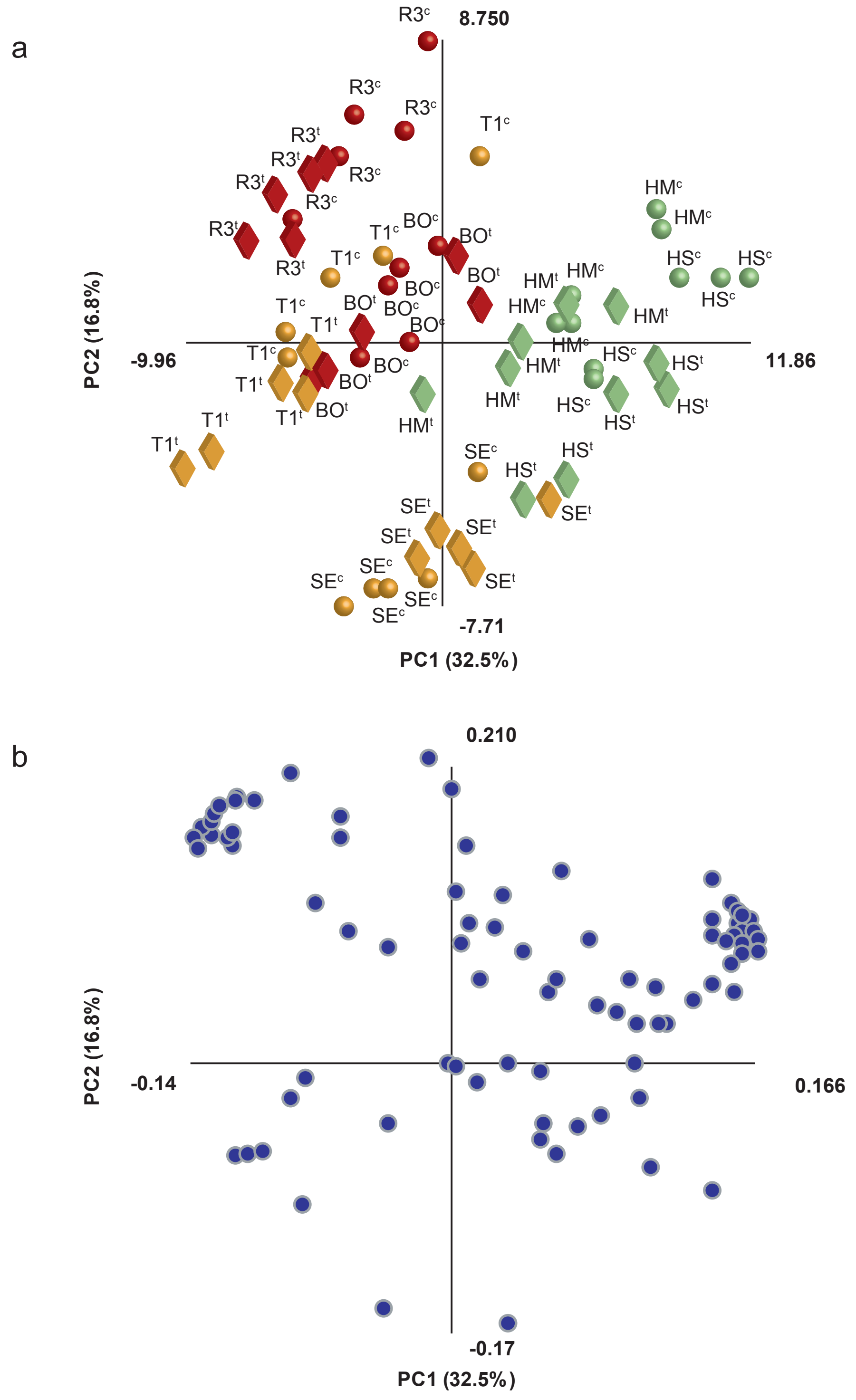

Fig. S4 Results of PCA analysis (a: scores plot, b: loadings plot) based on the metabolic profiles obtained through GC-MS analysis of 6 hop genotypes - HM, HS, T1, SE, R3, BO, see Table 1 for details on these genotypes - induced by aphid feeding (diamonds) or not (bullets). The experiment was performed in late summer (see Fig. S1).
