# Supplementary material for: The Use of Metabolomics to Elucidate Resistance Markers against Damson-Hop Aphid
Source: J Chem Ecol. 2018 Jul 6;44(7):711–26. doi: 10.1007/s10886-018-0980-y (PMC6096525; doi:10.1007/s10886-018-0980-y)
Supplement: Supplementary file 6 — (PDF 989 kb) [file 10886_2018_980_MOESM6_ESM.pdf]

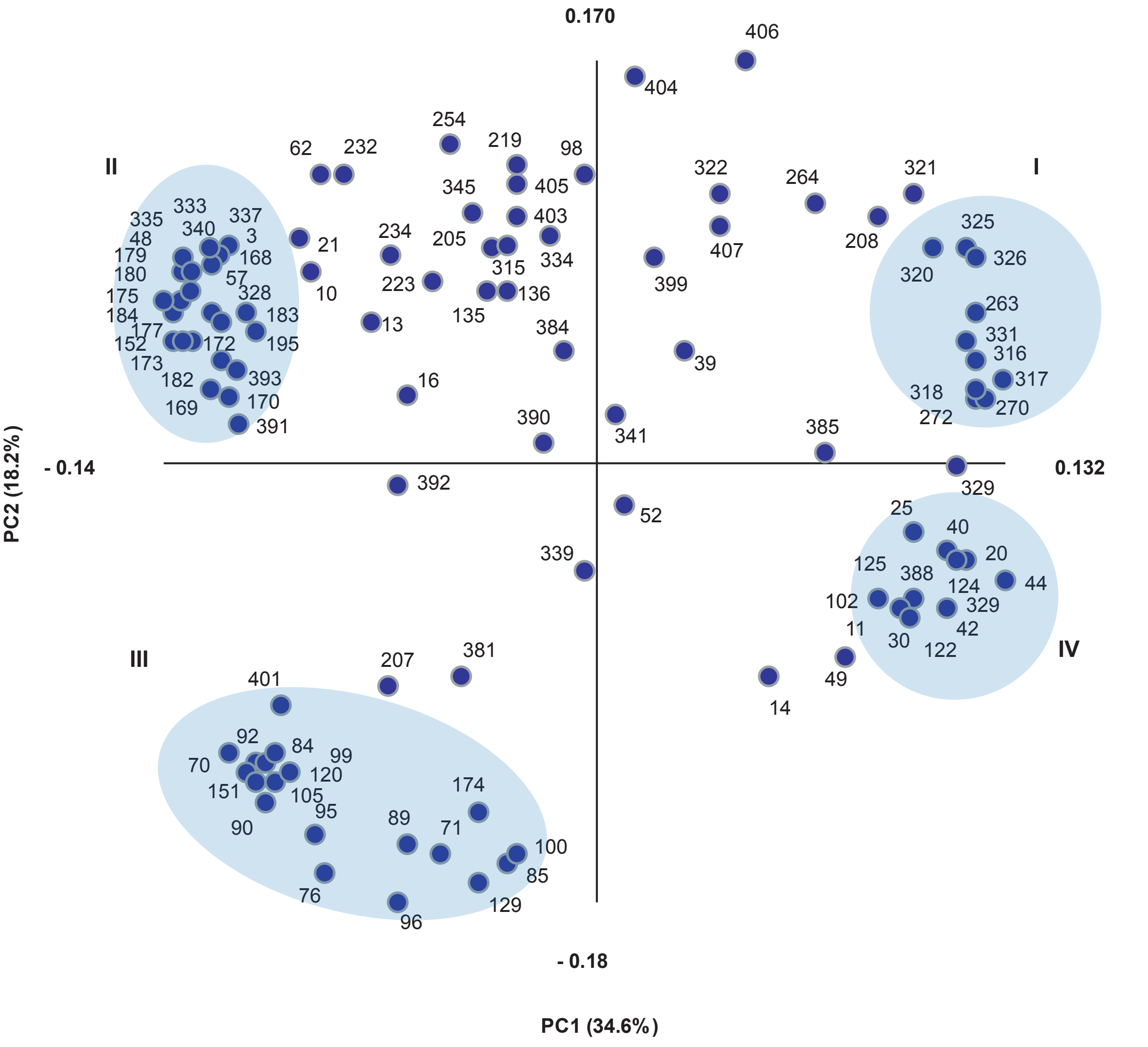

Fig. S5 PCA loadings plot representing the metabolic profiles obtained through GC-MS analysis of 6 hop genotypes with and without prior aphid feeding. Metabolites that drive the separation of hop genotypes into different resistance and aphid-induced or non-induced groups are highlighted in blue. The corresponding PCA scores plot is shown in Fig. 6.
